# Teaching the Ultrasound-Guided Fascia Iliaca Block to Emergency Medicine Residents: A Brief Educational Intervention

**DOI:** 10.7759/cureus.113375

**Published:** 2026-07-25

**Authors:** Caroline Molins, David Monaco, Soterios Stroud, Reshvinder Dhillon, Timothy Stokes

**Affiliations:** 1 Emergency Medicine, University of South Alabama Medical Center, Mobile, USA; 2 Emergency Medicine, University of South Alabama, Mobile, USA

**Keywords:** brief educational intervention, emergency medicine medical education, fascia iliaca compartment block, point-of-care-ultrasound, ultrasound-guided regional anesthesia

## Abstract

Introduction: The ultrasound-guided fascia iliaca block (FIB) has become an increasingly valuable skill in emergency medicine (EM) for managing acute pain in patients with hip and femur fractures. Despite its clinical utility, formal instruction on this block remains limited in EM training programs. This educational innovation describes a brief educational intervention (BEI) designed to teach EM residents the anatomy, technique, and indications for the ultrasound-guided FIB.

Methods: This novel curriculum combined an asynchronous pre-learning module with a focused simulation-based hands-on session. The asynchronous module included a 13-min instructional video as well as a pre- and post-test assessing knowledge of anatomy, indications, contraindications, and procedural steps. The in-person workshop consisted of residents rotating through hands-on ultrasound scanning stations to identify normal sonoanatomy and practice needle guidance using homemade ballistic gelatin models. A final course examination was given, as well as a post-course survey.

Results: A total of 15 EM residents at a university-based level I trauma center completed the program. Mean knowledge assessment scores improved from 58.9% pre-intervention to 78.4% post-intervention (p<0.01). On the post-course survey, of the nine residents who responded (60% response rate), all reported improved confidence and preparedness in performing ultrasound-guided FIBs.

Conclusion: This brief, reproducible, low-resource curriculum represents a feasible and effective strategy to integrate regional anesthesia training into EM residency ultrasound curricula.

## Introduction

The fascia iliaca block (FIB) is a regional anesthesia technique that provides analgesia for injuries involving the hip, femur, and anterior thigh by targeting the femoral and lateral femoral cutaneous nerves beneath the fascia iliaca compartment. It is an effective and safe alternative to systemic opioids, reducing pain scores, opioid consumption, and opioid-related side effects such as delirium and respiratory depression [1,2]. Increasing evidence supports its feasibility and safety when performed in the emergency department (ED), especially under ultrasound guidance [3].

Emergency physicians are uniquely positioned to perform this procedure, given their training in ultrasound-guided interventions and the growing emphasis on multimodal analgesia in acute care settings [4]. The American College of Emergency Physicians (ACEP) recognizes ultrasound-guided regional anesthesia as within the scope of emergency medicine (EM) practice. Integrating the FIB into ED workflows enhances patient-centered care, decreases reliance on opioids, and can improve throughput by facilitating imaging and orthopedic management [5].

However, despite evidence supporting its safety and efficacy, routine implementation of FIBs in the ED remains inconsistent [6]. Barriers include limited faculty expertise, a lack of standardized training models, and variability in access to ultrasound equipment [7]. Given the feasibility of teaching this block in short, structured sessions, there is an opportunity to increase EM resident competence and promote broader adoption of regional anesthesia. This educational innovation aimed to develop and evaluate a brief educational intervention (BEI) to teach EM residents ultrasound-guided FIBs through a combination of asynchronous learning and simulation-based practice.

## Materials and methods

A brief educational intervention was developed to teach emergency medicine residents ultrasound-guided fascia iliaca blocks during a three-year residency program at a level I trauma center in the Southeastern United States. The BEI was designed using spaced-repetition medical education theory as its conceptual framework, allowing learners to revisit procedural concepts through multiple educational exposures.

It consisted of the following two sequential components: an asynchronous learning module and a simulation-based hands-on workshop. The asynchronous component was delivered via the institution’s learning management system (Canvas; Salt Lake City, UT: Infrastructure, Inc.) and included a 13-min instructional video that reviewed regional anatomy, procedural indications, contraindications, complications, medication dosing, and step-by-step procedural technique. Residents completed a pre-education assessment prior to viewing the instructional material and a post-education assessment following completion of the module.

The second component consisted of an in-person simulation workshop facilitated by ultrasound-trained EM faculty. Residents rotated through ultrasound scanning stations to identify relevant sonoanatomy and practiced needle guidance techniques using homemade ballistic gelatin phantom models. Faculty provided brief demonstrations followed by deliberate practice with direct feedback. Each resident practiced the procedure at least once on each model during the approximately 40-min workshop. The spaced-repetition framework reinforced procedural learning through repeated exposure to core concepts during asynchronous instruction, knowledge testing, live scanning, and simulation-based procedural practice.

## Results

All 15 eligible EM residents completed both components of the BEI. Residents involved in curriculum development were excluded. Statistical analysis was performed using a paired-sample t-test to compare pre- and post-education assessment scores. Descriptive statistics were used to summarize survey responses and examination performance. Statistical analyses were conducted using Microsoft Excel (Redmond, WA: Microsoft Corporation). Statistical significance was defined as p<0.05.

Baseline pre-education assessments demonstrated limited familiarity with ultrasound-guided FIB concepts and procedural considerations (Table 1). Prior to the intervention, only 20% (3/15) of residents achieved a passing score of 75% or higher on the education assessment. Following completion of the BEI, 60% (9/15) achieved a passing score.

**Table 1:** Pre- and post-test course examination questions reported as number of residents who answered correctly out of 15 total residents. FIB: fascia iliaca block; n: number of residents who answered the question correctly; %: percentage of residents who answered the question correctly

Questions	Pre-education, n (%)	Post-education, n (%)
Fascia Iliaca blocks can be used for analgesia in which of the following patients?	4 (26.7)	11 (73.3)
It is important to document which of the following before and after the procedure?	10 (66.7)	9 (60)
What is the maximum dosage of bupivacaine that is used for the FIB?	9 (60)	12 (80)
How many mL of saline would you need to add to your bupivacaine to obtain the correct recommended total volume for injection when performing a FIB in a 75 kg patient?	0 (0)	9 (60)
After completion of a FIB, a patient complains of dizziness, numbness in the right upper arm, and confusion. HR is 55 bpm, and blood pressure is 95/65 mmHg. You believe the patient could be having...	15 (100)	14 (93.3)
Which of the following is the correct approach for a FIB?	12 (80)	13 (86.7)
The success of the nerve block is best predicted by?	10 (66.7)	12 (80)
A 62-year-old male with a closed femoral shaft fracture has an ultrasound-guided FIB performed. What sensory area of the leg does the FIB not provide relief to?	9 (60)	14 (93.3)
A FIB is performed with bupivacaine on a 10-year-old with a femur fracture. 6 h later, the patient reports being unable to feel the anterior thigh and knee, but strength is intact. What is the most likely cause?	11 (73.3)	12 (80)

Mean assessment scores improved from 58.9% pre-education to 78.4% post-education, representing a statistically significant improvement in procedural knowledge (paired t-test, t=2.98, p=0.0098) (Table 2). Improvement was observed across multiple content domains, including local anesthetic dosing, procedural documentation, recognition of expected post-block findings, and identification of complications such as local anesthetic systemic toxicity.

**Table 2:** Pre- and post-test performance of emergency medicine residents across all nine questions (n=15). The result is significant at p<0.05. n=15 for all data presented in this table.

Variables	Pre-education assessment	Post-education assessment
Mean test score, mean (%)±SD	58.9±14.3	78.4±19.44
Number of learners with passing score, n (%)	3 (20)	9 (60)

The largest improvements were observed in resident understanding of maximum safe bupivacaine dosing and appropriate procedural documentation. Correct responses regarding maximum bupivacaine dosage increased from 0/15 residents pre-course to 9/15 residents post-course. Similarly, the correct identification of required pre- and post-procedural documentation improved from 4/15 to 11/15 residents.

Two questions demonstrated a decrease in performance between pre- and post-test. A question regarding local anesthetic systemic toxicity decreased from 15/15 residents pre-course to 14/15 residents post-course. Similarly, a question regarding documentation requirements decreased from 10/15 residents pre-course to 9/15 residents post-course. The significance of these findings is unclear but may signify areas requiring further emphasis in course materials.

Following the intervention, residents also completed a post-education survey assessing procedural confidence and preparedness. Of the nine residents who completed the survey (60% response rate), all participants reported increased confidence in performing ultrasound-guided FIBs and felt more prepared to incorporate regional anesthesia into clinical practice. Participants additionally reported that the BEI improved their overall comfort with ultrasound-guided procedures and represented a valuable addition to EM residency education.

## Discussion

Ultrasound-guided regional anesthesia continues to gain importance within emergency medicine because of its role in opioid-sparing analgesia and patient-centered pain management. This curriculum improved both knowledge and confidence among EM residents in performing ultrasound-guided FIB. The findings mirror those of Paulson et al., who demonstrated that a 1-h rapid education event can successfully teach procedural ultrasound skills to physicians [8]. The success of this BEI lies in its hybrid approach of integrating pre-class asynchronous learning with a focused, high-yield simulation experience. This design aligns with adult learning theory and supports competency-based medical education principles by allowing residents to achieve procedural competence efficiently.

These results are particularly significant in the context of well-documented barriers to incorporating ultrasound-guided regional anesthesia (UGRA) into EM training. Wilson et al. surveyed emergency ultrasound leaders and found substantial variability in UGRA education across residencies [9]. While 93% viewed UGRA as an important resident skill, faculty expertise and experience were the primary barriers to implementation. Notably, 43% of faculty reported no formal UGRA training; over half were dissatisfied with their own knowledge; and programs with fewer than two ultrasound faculty members were significantly less likely to offer such training [9].

Additional studies corroborate these challenges, highlighting time constraints due to busy residency schedules, limited access to qualified instructors, the high costs of comprehensive workshops or commercial simulation models, interdepartmental resistance, and a lack of standardized protocols or credentialing pathways [10]. Comprehensive regional anesthesia workshops often require multi-day commitments, travel, multidisciplinary collaboration, and expensive resources, making them difficult to scale for most EM programs.

In contrast, our low-resource BEI directly addresses these barriers and offers a highly adoptable model. The low time requirement and minimal resource burden make this model easily adaptable for other programs seeking to expand procedural POCUS education. This BEI requires only a single point-of-care ultrasound (POCUS)-trained EM faculty member to lead the in-person sessions, standard ultrasound equipment, and affordable phantom models. Homemade models have been described in the literature using accessible supplies [11].

By demonstrating measurable improvements in resident knowledge and confidence through a brief, hybrid curriculum, this BEI contributes meaningfully to the existing literature on UGRA education. It serves as a practical bridge toward broader incorporation of regional anesthesia techniques into EM training programs. FIB is an ideal entry point procedure due to its relative simplicity, reproducibility, safety profile under ultrasound guidance, and strong evidence supporting opioid-sparing effects in hip and proximal femur fractures [12]. Wider adoption of such techniques aligns with national priorities for responsible opioid stewardship and may improve patient outcomes, including reduced opioid-related adverse effects such as delirium in vulnerable populations.

Overall, the significance of these findings lies in their potential to lower the previously described barriers for UGRA implementation in emergency medicine training programs. While prior work has primarily described barriers or resource-intensive solutions, this study provides a replicable, low-burden intervention that achieves meaningful educational gains. Scalable models like this BEI can help close the educational gap identified in national surveys, empower more EM providers to safely perform nerve blocks, and ultimately enhance acute pain management in the emergency setting.

Limitations

This educational innovation has several limitations. The sample size was small and derived from a single residency program, potentially limiting generalizability. Additionally, outcomes were limited to short-term knowledge acquisition and self-reported confidence; the intervention did not assess long-term skill retention. While increased confidence does not equate to clinical proficiency, these outcomes are appropriate for an initial curriculum innovation focused on feasibility and educational impact.

The study also did not evaluate downstream clinical outcomes, such as resident performance during patient care or effects on analgesic use in the emergency department. Future studies should include objective structured assessments, longitudinal follow-up, or multicenter implementation to better assess skill retention, clinical translation, and broader applicability of this curriculum.

## Conclusions

A brief educational intervention combining asynchronous learning with hands-on simulation significantly improved EM residents’ knowledge and confidence in performing ultrasound-guided fascia iliaca blocks. This streamlined, reproducible model provides a practical framework for integrating regional anesthesia training into EM residency curricula and supports the growing role of emergency physicians in providing advanced pain management through POCUS-guided techniques.
